# ﻿*Lysimachiapubiflora* (Primulaceae), a new species from Hubei, China

**DOI:** 10.3897/phytokeys.249.137900

**Published:** 2024-12-03

**Authors:** Han Xu, Song-Zhi Xu, Qi-Liang Gan, Zhen-Yu Li

**Affiliations:** 1 Chinese Academy of Inspection and Quarantine, Beijing 100172, China Chinese Academy of Inspection and Quarantine Beijing China; 2 School of Life Science, Nantong University, Nantong, Jiangsu 226019, China Nantong University Nantong China; 3 School of Pharmacy, Hubei University of Chinese Medicine, Wuhan 430072, China Hubei University of Chinese Medicine Wuhan China; 4 State Key Laboratory of Plant Diversity and Specialty Crops, Institute of Botany, Chinese Academy of Sciences, Beijing 100093, China Institute of Botany, Chinese Academy of Sciences Beijing China

**Keywords:** Hubei, *Lysimachiapubiflora*, new species, taxonomy

## Abstract

A new species, *Lysimachiapubiflora*, is described and illustrated from western Hubei Province, China. It is similar to *L.jinzhaiensis*, but differs in having flagelliform runners on the stems, indumentum on the plant and flowers, and the smaller calyx and anthers. The new species can be distinguished from all other species in the LysimachiaSubgen.LysimachiaSect.Nummularia by its glandular-pubescent corolla.

## ﻿Introduction

*Lysimachia* L. belongs to the family Primulaceae (*sensu lato*) (Angiosperm Phylogeny Group IV 2016; Sennikov 2016; Larson et al. 2023). This genus comprises approximately 180 species, mainly distributed in the temperate and subtropical regions of the Northern Hemisphere, with a few species found in Africa, Latin America, and Oceania (Chen and Hu 1979; Chen et al. 1989; Hu and Kelso 1996; Hao and Hu 2001). The “Flora of China” records that there are 138 species in China (Hu and Kelso 1996), primarily distributed in the southwestern Karst regions (Chen and Hu 1979).

In the past two decades, about 20 new endemic species have been discovered in China, predominantly in the expansive mountainous regions south of the Huai River (Yan et al. 2023; Zhang et al. 2024), highlighting Central China as a hotspot for *Lysimachia* diversity (Yi 2020; Ke et al. 2021). In June 2014, during an expedition to survey wild plant resources in Fang County, western Hubei Province, Qi-Liang Gan encountered an interesting *Lysimachia* species that resembles *Lysimachiahemsleyana* Maxim. ex Oliv. in having stems with whip-like branches (vs. terete stems and scattered glandular punctations on the leaf, calyx and corolla in *L.hemsleyana*). Further study showed that the newly collected species is more similar to *L.jinzhaiensis* S. B. Zhou & Kun Liu (Liu et al. 2014) both in the quadrangular stems, and scattered glandular striate on the leaf, calyx and corolla (vs. glabrous plant, wingless petiole, and without whip-like branches in *L.hemsleyana*). The diagnostic features distinguishing *L.hemsleyana*, *L.jinzhaiensis*, and *L.pubiflora* are summarized in Table 1. Based on unique combinations of characters, we propose that the newly collected specimens should be placed in LysimachiaSubgen.LysimachiaSect.Nummularia (Gilib.) Klatt (Chen et al. 1989), representing a species new to science.

**Table 1.** Morphological comparison among *Lysimachiajinzhaiensis*, *L.pubiflora*, and *L.hemsleyana*.

Characters	*L.jinzhaiensis*	*L.pubiflora*	*L.hemsleyana*
Stems	quadrangular, without flagelliform runners	quadrangular, with flagelliform runners	terete, with flagelliform runners
Indumentum on stems	glabrous, glandular on young part	pilose when young, otherwise glabrous	pilose
Petiole	narrowly winged, glabrous, not amplexicaul	narrowly winged, ciliate, amplexicaul	wingless, pilose, not amplexicaul
Leaf blade	1.5–5.5 × 1–4.5 cm, densely scattered glandular striate, both surfaces glabrous	1.5–9.5 × 1–6.5 cm, densely scattered glandular striate, adaxially glabrous, abaxially sparsely pubescent along the midrib when young	1.5–4 × 1.2–3 cm, densely scattered glandular punctate, adaxially densely, abaxially sparsely strigillose
Calyx lobes	narrowly ovate or elliptic-lanceolate, 6–8.5 × 3.5–4 mm, unequal, densely glandular striate, glabrous outside	elliptic or elliptic-lanceolate, 4.5–5 × 1.5–2 mm, unequal, densely glandular striate, sparsely pilose outside	narrowly lanceolate, 6.5–7.5 × 1–1.5 mm, subequal, densely glandular punctate, sparsely pubescent outside
Corolla	yellow, base orange-red; lobes elliptic, narrowly ovate to sublanceolate, 8–13 × 4–5.5 mm, densely glandular striate, glabrous	yellow, with an orange to orange-red base; lobes ovate-lanceolate, 10–12 × 2.5–3 mm, densely glandular striate, glandular-pubescent outside and along margins	yellow; lobes elliptic or elliptic-lanceolate, 4–6 × 3.5–4 mm, scattered glandular punctate, glabrous
Filaments	connate tube 3–4 mm long, free parts 3–5 mm long	connate tube ca. 2 mm long, free parts 4–6 mm long	connate tube ca. 2 mm long, free parts 3–5 mm long
Anthers	ca. 1.5 mm long	1.1–1.3 mm long	ca. 1.5 mm long

## ﻿Materials and methods

Specimens were collected in Fangxian County, Hubei Province. Comparisons were made with specimens of closely related species from main herbaria of China, such as PE, IBSC, HIB, KUN and several online databases, including CVH, JSTOR, IPNI, POWO, K, GH, P, and A (Holmgren et al. 1990; Fu 1993; Qin et al. 2019). All morphological characters were observed and measured using dissecting microscopes and described using the terminology suggested by Harris and Harris (1994).

## ﻿Taxonomic treatment

### ﻿ Lysimachia pubiflora

Taxon classificationPlantaeEricalesPrimulaceae

Q.L.Gan, Z.Y.Li & H.Xu sp. nov.

7F907E2E-0293-5BD0-A72A-27419352EF3A

urn:lsid:ipni.org:names:77352827-1

Figs 1 , 2

#### Diagnosis.

*Lysimachiapubiflora* is most similar to *L.jinzhaiensis* in several characteristics, including the quadrangular stems, glandular striations on the leaves, calyx, and corolla, as well as the presence of axillary solitary flowers featuring unequal calyx lobes. It also shares a yellow corolla with an orange-red base. However, *L.pubiflora* can be distinguished from *L.jinzhaiensis* by the presence of flagelliform runners on the stems (vs. absent in *L.jinzhaiensis*), pilose young stems and calyx lobes (vs. glabrous in *L.jinzhaiensis*), calyx lobes 4.5–5 mm long (vs. 6–8.5 mm long), and anthers 1.1–1.3 mm long (versus ca. 1.5 mm long). The new species can easily be distinguished from all other species in Sect. Nummularia by its glandular-pubescent corolla.

**Figure 1.**
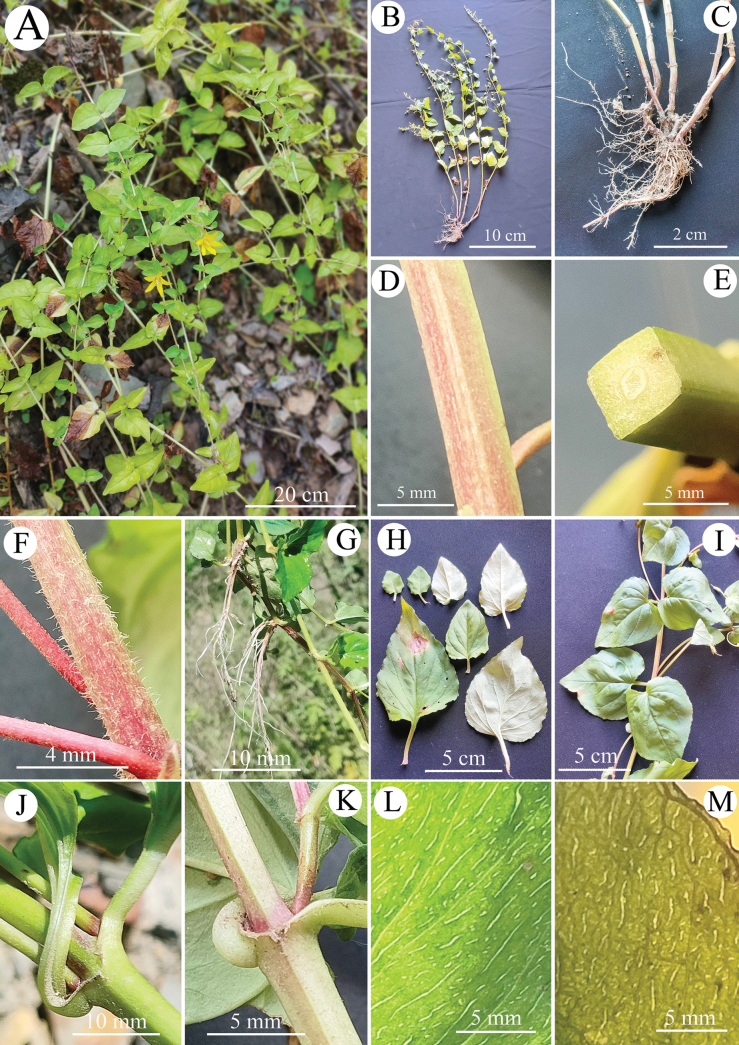
*Lysimachiapubiflora* sp. nov. **A** plant at early flowering stage **B** mature individual **C** rhizome and adventitious roots **D** stem **E** cross section of stem **F** pubescent young stem **G** upper part of flagelliform runner, showing the distal nodes with adventitious roots **H** leaves **I** larger leaves with broad-cordate base **J, K** petioles **L, M** glandular stripes on leaf blades usually transparent (**L** when fresh, **M** dried).

#### Type.

China • Hubei Province, Fangxian County, Hongta Town, Nantang Village, alt. 712 m, 12 June 2024, *Qi-Liang Gan 4450* (holotype, PE !).

***Paratypes*.** China • Hubei: Fangxian County, Yerengu Town, Tanjiawan Village, alt. 697 m, 12 June 2024, Qi-Liang Gan 4447 (PE !) • Fangxian County, Yerengu Town, Tanjiawan Village, alt. 697 m, 12 June 2024, Qi-Liang Gan 4448 (PE !) • Fangxian County, Hongta Town, Nantang Village, alt. 712 m, 12 June 2024, Qi-Liang Gan 4449 (PE !).

**Figure 2.**
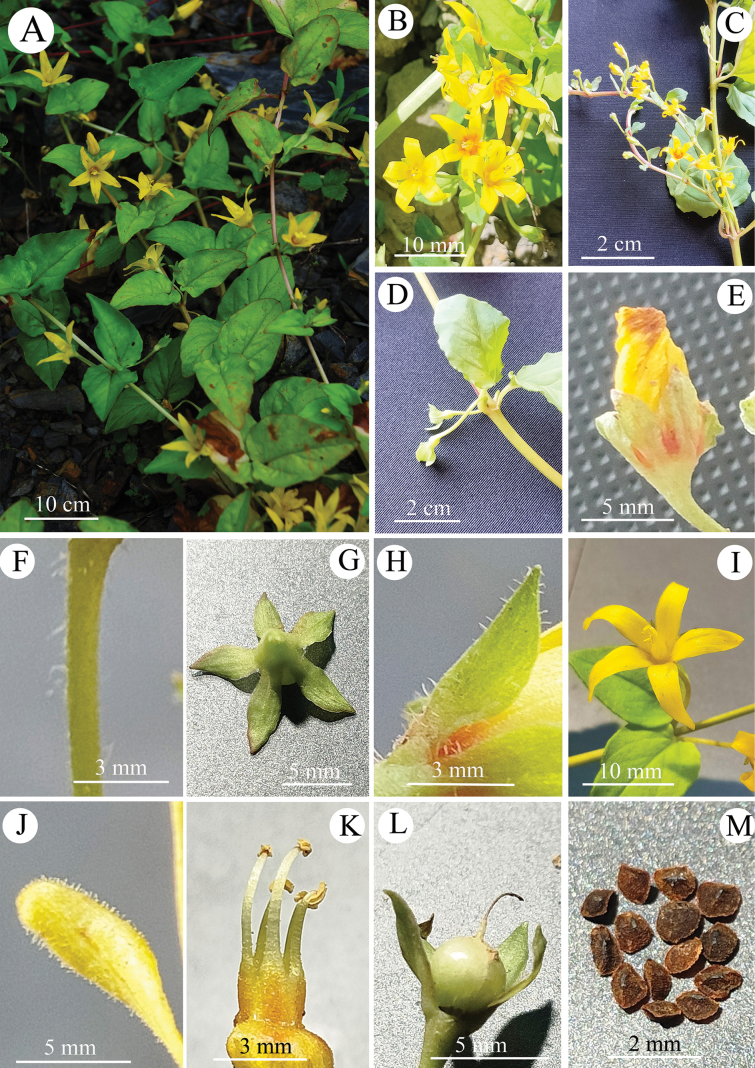
*Lysimachiapubiflora***A** plants at full-bloom **B, C** flowering branches **D** axillary flowers **E** flower bud **F** pedicel **G** calyx (adaxial view) **H** calyx (lateral view) **I** corolla **J** a lobe of corolla (abaxial view) **K** stamens **L** capsule with persistent calyx **M** seeds.

#### Description.

Herbs perennial. Rhizome horizontal, below-ground, 5–12 cm long, with adventitious roots at the nodes; stems usually 3–5 caespitose, 50–120 cm long, quadrangular, pubescent, at least when young, with a spreading habit, distal parts of stems and branches usually slender and smaller leaved, forming the flagelliform runners that usually root at the 1–3 distal nodes. Leaves opposite, rarely 3-whorled in the upper section of stems; petioles 0.5–2.5 cm long, adaxial sides shallowly grooved, abaxial sides rounded, narrowly winged, margins pilose, amplexicaul at the base; leaf blades broadly ovate to deltoid-ovate, 1.5–9.5 cm long, 1–6.5 cm wide, with acute or subobtuse apices, broadly cordate, subrounded or truncate, rarely cuneate at the base (on the runners), margins entire or slightly undulate, densely scattered with transparent glandular striations that sometimes turn purple when dry, glabrous adaxially, and abaxially sparsely pubescent along the midrib when young, becoming glabrate with age; lateral veins in 4–6 pairs, with the lowest 1–2 pairs arising from the base, the others alternating, midrib and lateral veins impressed adaxially, raised adaxially, veinlets inconspicuous. Flowers solitary in axils of leaves; pedicel 1.1–2.5 cm long, usually shorter than subtending leaves, sparsely pubescent. Calyx 5–5.5 mm long, 5–parted almost to the base, connate part ca. 0.5 mm, lobes elliptic or elliptic-lanceolate, unequal, 4.5–5 × 1.5–2 mm, densely transparent glandular striate, the stripes becoming purple when dry, sparsely pilose outside; corolla yellow, with an orange or orange-red base, rotate, 2–2.3 cm in diam., 5-parted, tube 1–1.5 mm long, lobes narrowly lanceolate, 10–12 mm long, 2.5–3 mm wide, sparsely transparent glandular striate, the stripes becoming black-purple when dry, glabrous inside, glandular-pubescent outside and along margins; stamens 5, adnate to the base of the corolla tube, erect, yellow, glabrous, filaments basally connate into a tube ca. 2 mm long, free parts 4–6 mm long, anthers basifixed, oblong, 1.1–1.3 mm long, open by lateral slits; pistil glabrous, ovary globose, ca. 1.5 mm in diameter, the style filiform, 7–8 mm long, stigma obtuse, slightly wider than the style. Capsule subglobose, 3–4 mm in diam., glabrous. Seeds dark brown, rhombic, 0.5–1 mm long, 3–4 angled, glabrous.

#### Phenology.

Flowering from late May to early July; fruiting from mid-July to late August.

#### Distribution and habitat.

This species is endemic to two specific townships in Fang County, confined to a narrow limestone valley that lies between the neighboring villages of Tanjiawan and Nantang. It is observed along roadsides, within water ditches, in sparse shrublands on hillsides, and at the edges of sparse forests. The elevation of its habitat ranges from 690 to 712 meters.

The main companion plant species include trees such as *Populusadenopoda* Maxim., Quercusserratevar.brevipetiolata (A. DC.) Nakai, *Platycaryastrobilacea* Sieb. & Zucc., *Broussonetiapapyrifera* (L.) L’Hér. ex Vent., *Verniciafordii* (Hemsl.) Airy Shaw, *Pinusmassoniana* Lamb.; shrubs such as Cotinuscoggygriavar.pubescens Engl., *Mallotusapelta* (Lour.) Müll. Arg., *Buddlejaofficinalis* Maxim., *Pyracanthafortuneane* (Maxim.) Li, *Zanthoxylumarmatum* DC., *Coriarianepalensis* Wall., *Rhuschinensis* Mill., *Salixwallichiana* Anderss., *Indigoferabungeana* Walp., *Ficusheteromorpha* Hemsl., *Linderaglauca* (Sieb. & Zucc.) Bl., Rosabanksiaevar.normalis Regel. Herbaceous plants consist of *Miscanthusfloridulus* (Lab.) Warb. ex Schum. & Laut., *Anemonehupehensis* Lem., Geumjaponicumvar.chinense F. Bolle, *Agrimoniapilosa* Ledeb., *Duchesneaindica* (Andr.) Focke, *Asteralbescens* (DC.) Hand.-Mazz., *Leersiajaponica* (Makino) Honda, Pteridiumaquilinumvar.latiusculum (Desv.) Underw. ex A. Heller, *Pterisvittata* L., *Cyrtomiumtsinglingense* Ching & K. H. Shing ex K. H. Shing, and others. Vines include *Clematisarmandii* Franch., *Biancaeadecapetala* (Roth) O. Deg., *Dalbergiamimosoides* Franch., and *Smilaxglauco-china* Warb.

#### Etymology.

The epithet ‘pubiflora’ refers to the glandular-pubescent corolla. Vernacular name: Mao Hua Guo Lu Huang (Chinese).

#### Conservation assessment.

This species inhabits a narrow limestone valley, extending approximately ten kilometers in straight-line distance between two villages. The region is characterized by significant limestone exposure and thin, infertile soil layers, reflecting a fragile natural ecosystem. This ecosystem is highly susceptible to human activities. Following its initial discovery in 2014, the species has shown significant population fragmentation due to road construction, deforestation for agriculture, and livestock grazing. The current population size is estimated to consist of around one thousand individuals. Based on the IUCN Guidelines (Version 16) (IUCN 2024), the species may be classified as ‘Endangered’.

## Supplementary Material

XML Treatment for Lysimachia pubiflora

## ﻿Additional information

### Author ORCIDs

Han Xu https://orcid.org/0000-0001-9056-4173

Song-Zhi Xu https://orcid.org/0000-0002-4178-5559

### Data availability

All of the data that support the findings of this study are available in the main text.
